# Poorly Differentiated Large Cell Neuroendocrine Carcinoma of the Colon: A Case Report

**DOI:** 10.7759/cureus.20949

**Published:** 2022-01-05

**Authors:** Kelsee Felux, Burke McCarty, Dennis Turner, TKeyah Gray, Vijaykumar Patel

**Affiliations:** 1 General Surgery, Wellstar Atlanta Medical Center, Atlanta, USA

**Keywords:** neuroendocrine carcinoma(nec), neuroendocrine carcinoma colon, large cell neuroendocrine, invasive colon cancer, colon resection, extended right hemicolectomy, right side colon carcinoma, poorly differentiated neuroendocrine carcinoma

## Abstract

Colon cancer is one of the most common diagnoses of cancer and a leading cause of death in America. Large cell neuroendocrine tumors are a very uncommon type of colon cancer that tends to have a poor prognosis. Usually, these tumors are only found at the time of metastasis making them even more difficult to treat. A 65-year-old female presented with worsened generalized abdominal pain associated with abdominal distention. She had not had a bowel movement in over a week and did not have any flatulence. She had a colonoscopy four years prior that was normal. Physical examination was significant for abdominal distention and a large right-sided palpable mass in her abdomen with generalized tenderness. A CT scan showed a large irregular mass at least 9.8 x 10.5 cm at the mid to distal ascending colon resulting in significant colonic narrowing significant for a large bowel obstruction. The CT also demonstrated suspicious nodules in the lung, lesions in the liver, and lymphadenopathy. She had an exploratory laparotomy with an extended hemicolectomy to remove the mass. Pathology revealed the mass was neuroendocrine carcinoma, a large cell subtype, that was poorly differentiated with involvement of at least 32 of 34 lymph nodes. This tumor was positive for AE1/AE3, CEA, CK20, and synaptophysin. Ki-67 showed 70% positivity. TTF1 was negative and ruled out a primary lung tumor. Microsatellite immunostains were positive for MLH-1, MSH-2, MSH-6, and PMS2. The patient was started on Carboplatin AUC6 and Etoposide 100mg/m^2^ in three-week intervals. Pegfilgrastim was also added to her treatment plan every 21 days. This is a review of a female who presented with colonic obstruction that was found to be poorly differentiated large cell neuroendocrine carcinoma after a previous negative colonoscopy.

## Introduction

Learning objectives

- Common locations of neuroendocrine carcinomas, epidemiology of neuroendocrine carcinomas of the colon, and how aggressive and fast these metastasize.

- Treatment options for neuroendocrine carcinomas of the colon. 

- Current guidelines of screening for colon cancer and how some tumors may still appear after a negative colonoscopy.

- Review of common symptoms of left versus right-sided colon cancers.

- Explain the importance of fully working up someone with iron deficiency anemia that may be at high risk for colon cancer.

- Describe how Ki-67 is used as a predictor of aggressiveness of these tumors.

Neuroendocrine Tumor (NET) is a rare tumor with only approximately 12,000 people in the United States being diagnosed each year, and <200,000 people currently living with this tumor [1]. It accounts for 0.5% of all malignancies and only 2% of tumors in the gastrointestinal tract [2]. There was a study done by Bernick et al. that found that of the patients that had colorectal cancer 0.6% of them were found to be NET, and of 0.6% of cases only 0.2% of them were found to be large cell NETs (LCNETs) [3]. The histological features of LCNETs are characterized by (i) neuroendocrine appearance under light microscopy - organoid, nesting, trabecular, rosette, and palisading pattern, (ii) large cells with a polygonal shape, ample cytoplasm, coarse chromatin, and frequent nucleoli, (iii) very high mitotic rate along with frequent of necrosis and evidence of neuroendocrine features by immunohistochemistry or electron microscopy [4]. Ki-67 index and the mitotic count are used for classification into categories from G1-G3 [5]. Poorly differentiated Neuroendocrine Carcinomas (NECs) are now either large- or small-cell and are classified as G3 NECs. Additionally, carcinomas are classified as NEC G3 with a high mitotic rate >20 per 10 high-power fields (HPF) and Ki-67 labeling index of >20% [5]. Tumors that fall into this category are rare, extremely aggressive, and usually metastasize in the early stages. [5]. By the time these tumors are found they have most likely metastasized to multiple organs with the most common location being the liver [6]. We present a case of a 65-year-old female with a metastatic LCNET of the colon who presented with sharp, generalized, abdominal pain that was worsening for three days.

## Case presentation

A 65-year-old African American female with a past medical history of hypertension, hyperthyroidism, hyperlipidemia, type 2 diabetes, and obesity presented to the emergency department with worsened generalized abdominal pain onset two to three days ago associated with abdominal distension. She noticed intermittent sharp abdominal pain for about one month and abdominal distension for one week prior. The pain worsened with meals and movement and was relieved with rest. She reported constipation and her last bowel movement was over one week ago. She has noticed a decreased appetite, nausea, abdominal distension, and lack of flatulence. She denied diarrhea, fever, chills, and vomiting. Four months ago, she was seen at another hospital for chest pain. Her hemoglobin was 5.5 g/dL, and she was given a blood transfusion of two units. After the blood transfusion, her chest pain symptoms improved, and she was told to follow up outpatient with a prescription for ferrous iron. She has a surgical history of a hysterectomy for fibroids, tubal ligation, and thyroid surgery. She had a colonoscopy done four years prior that showed normal mucosa with no masses, polyps, or ulceration. She has a family history of hypertension, heart disease, and stroke. She does not smoke any cigarettes, denies drug use, or alcohol. Her medications included aspirin, metformin, insulin, losartan, amlodipine, hydrochlorothiazide, and atorvastatin. She had no known allergies.

**Figure 1:**
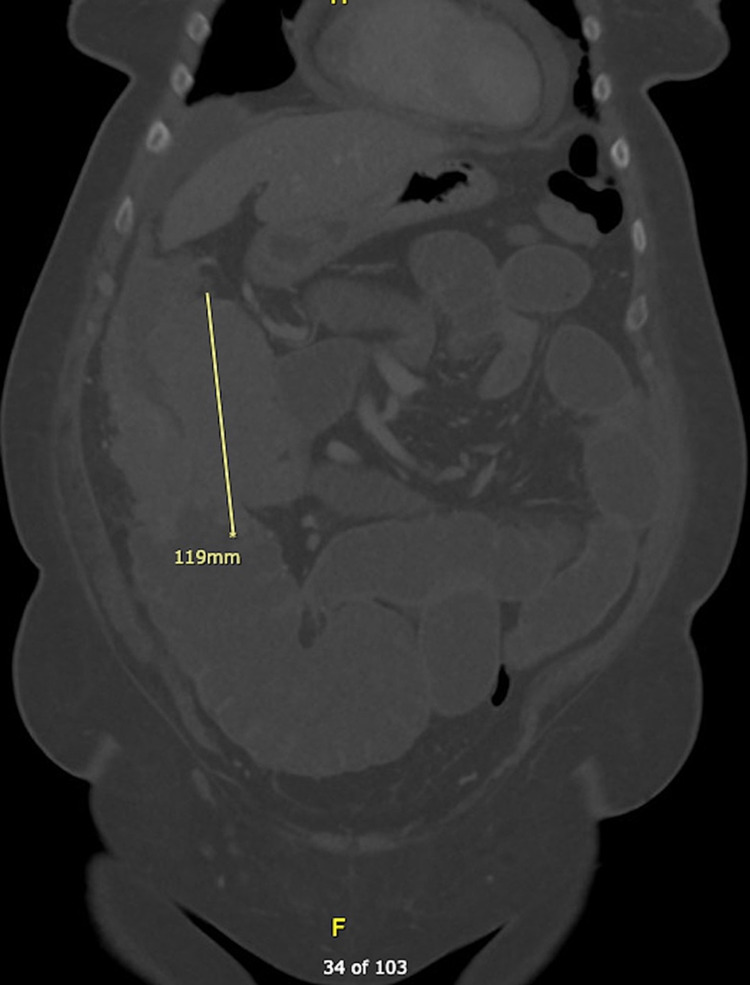
Computed tomography, coronal view, revealing an irregular mass at the mid to distal ascending colon with signs of obstruction.

**Figure 2:**
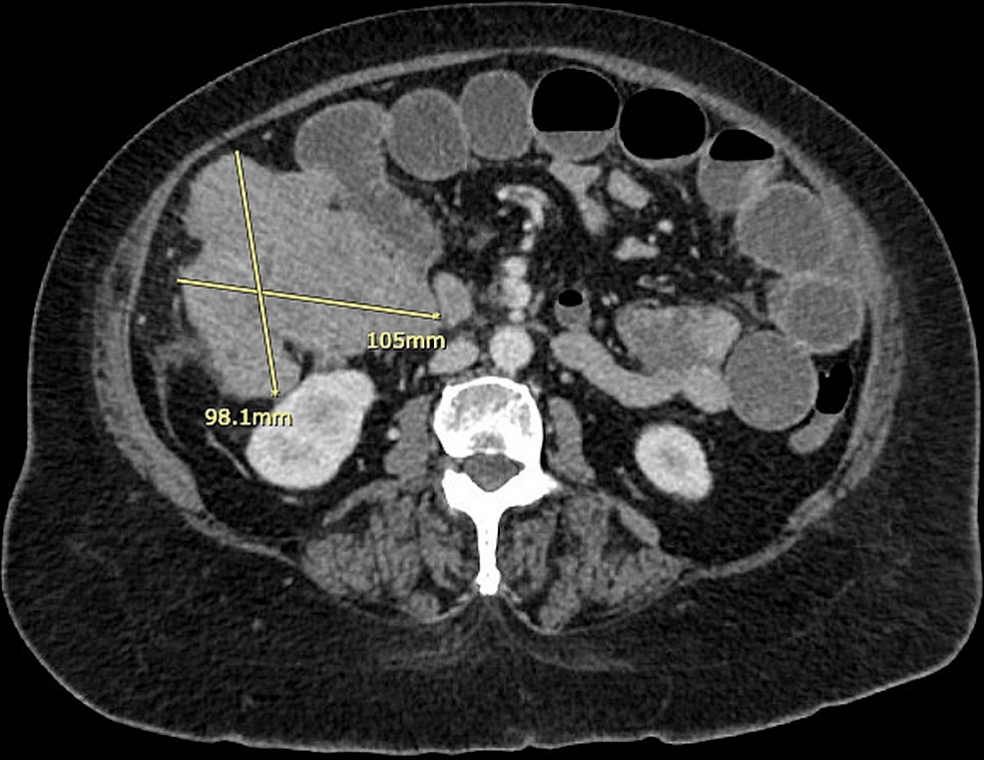
Computed tomography, cross-sectional view, showing the large right colon mass causing obstruction.

On physical examination, the patient was hypertensive at 176/98 mmHg, tachycardic at 107 bpm, and afebrile at 98.4F. Her respiratory rate was 17 bpm and SpO2 was at 94% on room air. She appeared obese but she was in no acute distress and did not appear ill. Her cardiovascular and respiratory examinations were normal. Her abdomen was soft and distended. There were decreased bowel sounds, generalized abdominal tenderness, and no peritonitic signs. A large mass was palpable on the right side of the abdomen. Laboratory evaluation was significant for a white blood cell count at 11.33 x 10^9^/L (3.50-10.50 x 10^9^/L). The hemoglobin was 9.0 g/dL (12.0-15.5 g/dL), hematocrit 31.5% (35%-45%), MCV 66.9 fL ​​(82.0-98.0 fL), MCHC 28.6 g/dL (32-36 g/dL). The RDW was 19.7% (11.9%-15%) and platelet count was 484 x 10^9^/L (150-450 x 10^9^/L). On the basic metabolic panel, all electrolytes, renal, and liver values were normal. Glucose was 117 mg/dL (70-105 mg/dL) and globin was 3.5 g/dL (1.5-3.0 g/dL). Lipase was 9 U/L (11-82 U/L). A flat and upright x-ray of the abdomen with chest revealed mild prominence of the hila with no lung consolidation and no pleural effusion or pneumothorax. There was a nonspecific bowel gas pattern with large and small bowel present. Gas-filled small bowel loops in the left abdomen approached four to five centimeters in transverse dimension. The CT of the abdomen and pelvis with IV contrast showed a large irregular mass that measured at least 9.8 x 10.5 cm at the mid to distal ascending colon. This resulted in significant colonic narrowing and large bowel obstruction with fluid distension of the small bowel loops proximal to the location (see Figures 1, 2). There was a right hepatic lobe lesion measuring 1.5 x 1.1 cm and a left adrenal lipoma or myelolipoma measuring 1.3 x 1.1 cm. Bilaterally there were randomly distributed pulmonary nodules seen. A left lower lobe pulmonary nodule measured 1.2 x 1.7 cm, a right lower lobe nodule measured 1.2 x 1.0 cm, and a right subpleural nodule measured 0.8 x 1.0 cm. A moderate complex pericardial effusion and ascites were found.

**Figure 3:**
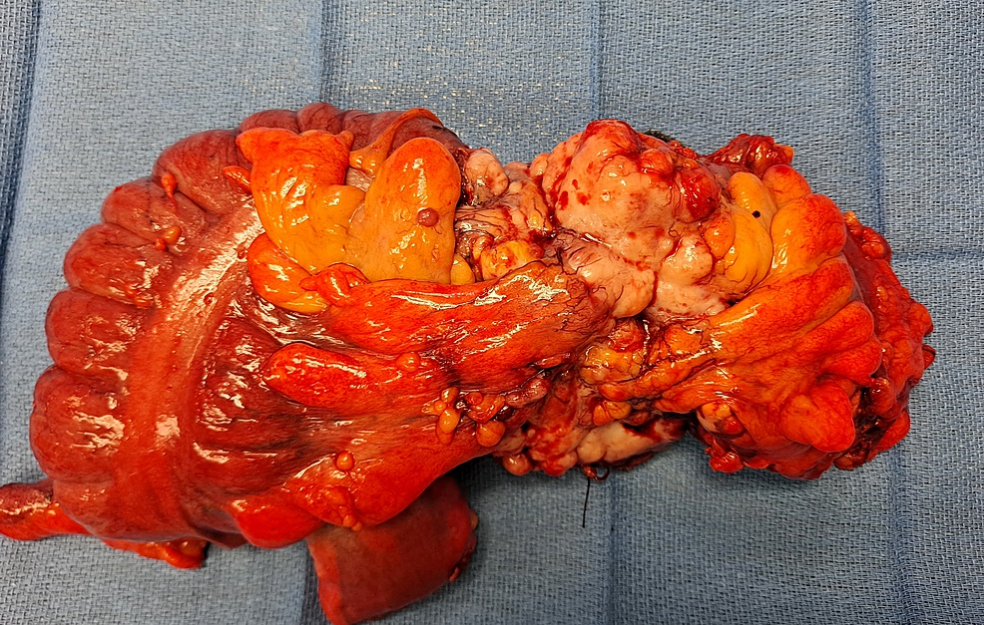
The gross image of the resected irregular mass in the right colon with extension into surrounding structures.

Further laboratory tests were ordered, general surgery was consulted for the bowel obstruction and mass, and cardiology was consulted for the pericardial effusion. Tumor marker studies showed Ca 19-9 was 133 U/mL (<34 U/mL), Ca 125 was 24 U/mL (<35 U/mL), and CEA was >1,021 (0.0-3.0 ng/mL). A TSH was measured at 3.26 µLU/mL (0.34-5.60 µLU/mL). She had an echocardiogram that showed an ejection fraction of 61%-65%. There was a moderate pericardial effusion with no evidence of tamponade. After the consent of the patient, an exploratory laparotomy revealed a large mass in the right colon with extension into the mesentery, liver, colon, and perinephric tissue posteriorly (Figure 3). An extended right colectomy with anastomosis, mass debulking, liver biopsy, left oophorectomy, right salpingo-oophorectomy, and mesenteric biopsy was performed by general surgery and sent to pathology. She did not require an ileostomy. Post-operatively she required a transfusion. She was discharged with no complications once stabilized.

**Figure 4:**
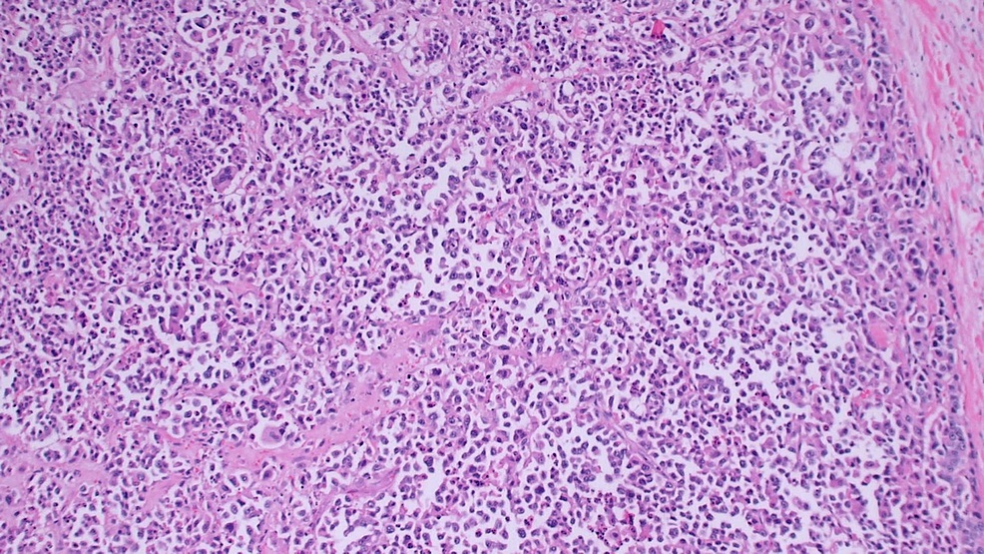
Microscopic image of organoid growth pattern with some nesting of cells resembling large cell carcinoma.

The surgical pathology report showed poorly differentiated, large cell, neuroendocrine carcinoma of the colon measuring 9.5 x 10 cm with the full thickness of the muscularis propria to the subserosa. Proximal and distal margins were negative, but radial margins were positive. Metastatic carcinoma was found in the mesenteric lymph nodes (32 out of 34), liver segment, and retroperitoneal lymph node. The portion of the ovary and fallopian tube that was removed was negative for neoplasm. Microscopic evaluation revealed an organoid growth pattern with some nesting of cells with moderate nuclear atypia and moderate amounts of generally pinkish cytoplasm (Figure 4). Due to these unusual features, a battery of special studies was performed.

**Figure 5:**
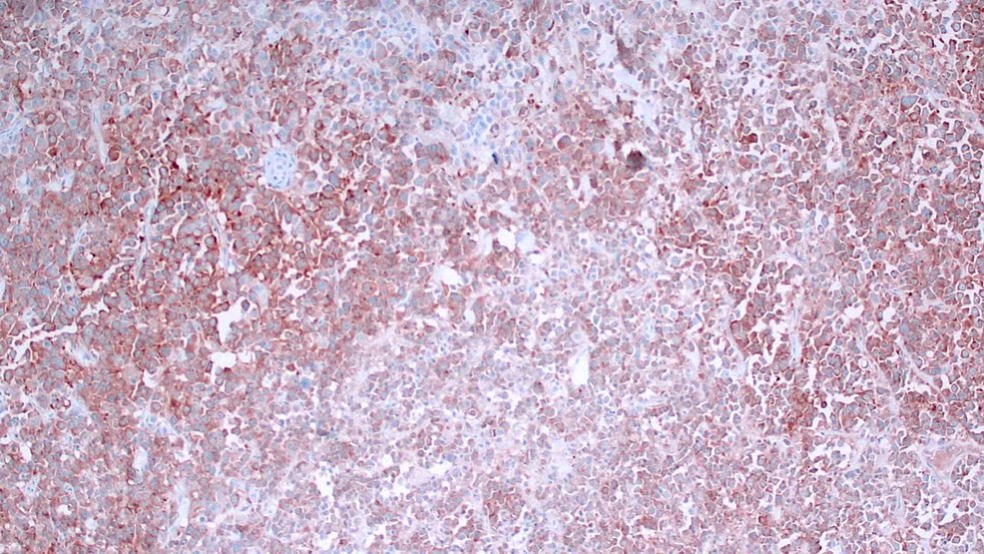
Staining positive for synaptophysin.

Flow cytometry was performed to assess the possibility of lymphoma but found insufficient viable B cells for reliable clonality assessment as the sample showed very low viability of only 3.5%. Immunostains showed the tumor to be positive for AE1/AE3, CEA, CK20, and synaptophysin (see Figure 5).

The tumor was negative for AFDP, CD56, glypican-3. There was a patchy minimal positively with NSE. MUC2 and MUC5AC show patchy weak positivity. CK7 shows patchy positivity. Chromogranin was negative. CDX2 showed equivocal positivity. Ki-67 showed 70% positivity. To rule out primary lung tumor TTF1 was ordered and was negative. Microsatellite immunostains were positive for MLH-1, MSH-2, MSH-6, and PMS2. This was consistent with intact nuclear expression of mismatched repair proteins and low probability of MSI-H. She was started on treatment with Carboplatin ACU 6 and Etoposide 100mg/m^2^. Pegfilgrastim was added every 21 days. 

## Discussion

Large cell neuroendocrine carcinoma of the colon is rare and often extremely aggressive with metastatic disease at the time of initial diagnosis. Even with treatment, the prognosis of these tumors is exceedingly poor. A study by Bernick et al. showed that 0.6% of patients with colorectal cancer had neuroendocrine carcinoma and only 0.2% of those were large cell neuroendocrine carcinomas [3]. Most NETs arise in the gastrointestinal tract and respiratory tract. Of the gastrointestinal tract, the common locations include the small intestine (38%), rectum (34%), large intestine (16%), stomach (11%), and unknown sites (1%) [7]. In addition to our patient having an extremely rare tumor, the location is also unique. Her tumor presented in the large intestine while most arise in the small intestine or the rectum. First-line treatment for large cell neuroendocrine carcinomas is surgery to remove the tumor [6]. The role of surgery was to relieve the obstruction and decrease the amount of tumor to improve survival. These are then treated with a combination of a platinum-based agent, Cisplatin or Carboplatin, in combination with Etoposide [8]. There is very limited data on the use of second-line agents and further effective and safe options should be further explored. 

Several screening tests are used for colon cancer including fecal testing, sigmoidoscopy, and colonoscopy with colonoscopy being the most common [9]. According to the American Cancer Society, current guidelines suggest a colonoscopy every 10 years after a normal test starting at 45 years old for the patient at average risk of developing colon cancer [10]. More aggressive tumors may occur quicker than in a 10-year time. Singh et al. found that right-sided cancers were more likely to occur in patients with a negative initial colonoscopy. This study showed that colon cancer cases are most likely to be right-sided in the initial two years post a negative colonoscopy compared to five years [11]. This is important in our patient as she had a normal colonoscopy four years ago. Furthermore, a colonoscopy may be better for finding left-sided tumors than right-sided tumors [9]. Physicians and researchers may need to find an improved screening method for neuroendocrine and right-sided tumors of the colon to improve overall detection and survival rates in these patients. Current guidelines do not address rapidly dividing tumors that are missed within the ten years [9].

A study by Saidi et al. showed that right-sided colon cancers are known to decrease hemoglobin and are less likely to present with obstruction, abdominal pain, and altered bowel habit [12]. Symptoms of the left side of the colon are usually more obvious and generally include rectal bleeding, constipation, abdominal pain, and obstruction [12]. Our patient presented with obstruction, abdominal pain, and anemia, with the first two symptoms being more indicative of left-sided tumors. A study by Goodwin and Irvin showed that in patients with right-sided tumors the most common error was a failure to initiate or fully investigate patients with iron deficiency anemia [13]. Our patient also had iron deficiency anemia that was diagnosed at another institution when she presented with chest pain. After a transfusion, her chest pain and symptoms improved, and she was sent home to follow up with a CT scan that was never completed. This demonstrates the importance of further investigation of iron deficiency anemia due to the possibility of cancer. More importantly, even with a negative colonoscopy within 10 years, the diagnosis of iron deficiency anemia should not be overlooked in at-risk populations. Physicians should also emphasize the importance and ensure that patients have an understanding of why follow-up diagnostic studies are important during physician-patient discussions.

Pascarella et al. described a case report reinforcing the aggressive nature of colonic LCNETs being evident in their case by the rapid progression of the tumor and near replacement of the liver by the tumor in a period spanning approximately 10 days from the initial presentation to the time of second operative exploration [6]. Pascarella’s patient had an initial CT of the abdomen and pelvis showing only a lesion in the right colon with a dilated cecum and small bowel and just 10 days post-operative a comparable CT scan showed a hepatic lesion and later confirmed metastatic large cell neuroendocrine carcinoma [6]. This demonstrates how rapid the spread of NETs occurs and how metastasis has already ensued on initial presentation. Our patient had a most recent colonoscopy done just four years prior without any evidence of masses, polyps, or ulceration. This cancer progresses in a fast nature and most likely will have metastasized to multiple organs by the time it is found. This provides support that these tumors need to be detected earlier to possibly improve survival rates in these patients.

The aggressiveness of NETs can be distinguished by a combination of microscopy which analyzes the cellular structures and immunohistochemical staining of the cell cycle antigens. Ki-67 is an antigen marker for cell proliferation and is detected in the nucleus of actively cycling cells [14]. The tumor described in this case demonstrated 70% of the tumor cell nuclei staining positive for Ki-67 with it measuring 9.5 x 10 cm validating its excessive growth and aggressiveness with a rapid metastasis to distant organs. The different methods of determining the Ki-67 index include manual counting, eyeballing, or digital imaging analysis [15]. Manual counting is thought to be too time-consuming and imaging analysis is not readily available to the general population. Eyeballing is the current method of choice, but the variability is high between different observers. This can cause a percentage difference from one grade to another [15]. This method may lead to a lack of precision between multiple patients.

## Conclusions

In conclusion, LCNETs of the colon are very uncommon with a poor prognosis due to their aggressive nature. At the time these tumors are found, they have usually metastasized to multiple organs. This causes challenges in treatment and decreased survival rates. Earlier detection may help provide a better prognosis for future patients. Anemia in high-risk patients should raise more concern for cancer even with a negative colonoscopy within 10 years.

## References

[1] Dasari A, Shen C, Halperin D (2017). Trends in the incidence, prevalence, and survival outcomes in patients with neuroendocrine tumors in the United States. JAMA Oncol.

[2] Moertel CG (1987). Karnofsky memorial lecture. An odyssey in the land of small tumors. J Clin Oncol.

[3] Bernick PE, Klimstra DS, Shia J (2004). Neuroendocrine carcinomas of the colon and rectum. Dis Colon Rectum.

[4] Travis WD, Linnoila RI, Tsokos MG (1991). Neuroendocrine tumors of the lung with proposed criteria for large-cell neuroendocrine carcinoma. An ultrastructural, immunohistochemical, and flow cytometric study of 35 cases. Am J Surg Pathol.

[5] Klimstra DS, Arnold R, Capella C (2010). Neuroendocrine neoplasms of the pancreas. WHO Classification of Tumours of the Digestive System.

[6] Pascarella MR, McCloskey D, Jenab-Wolcott J, Vala M, Rovito M, McHugh J (2011). Large cell neuroendocrine carcinoma of the colon: a rare and aggressive tumor. J Gastrointest Oncol.

[7] Yao JC, Hassan M, Phan A (2008). One hundred years after "carcinoid": epidemiology of and prognostic factors for neuroendocrine tumors in 35,825 cases in the United States. J Clin Oncol.

[8] Strosberg JR, Coppola D, Klimstra DS, Phan AT, Kulke MH, Wiseman GA, Kvols LK (2010). The NANETS consensus guidelines for the diagnosis and management of poorly differentiated (high-grade) extrapulmonary neuroendocrine carcinomas. Pancreas.

[9] Rex DK, Johnson DA, Anderson JC, Schoenfeld PS, Burke CA, Inadomi JM (2009). American College of Gastroenterology Guidelines for colorectal cancer screening 2008. Am J Gastroenterol.

[10] Wolf AM, Fontham ET, Church TR (2018). Colorectal cancer screening for average-risk adults: 2018 guideline update from the American Cancer Society. CA Cancer J Clin.

[11] Singh H, Turner D, Xue L, Targownik LE, Bernstein CN (2006). Risk of developing colorectal cancer following a negative colonoscopy examination: evidence for a 10-year interval between colonoscopies. JAMA.

[12] Saidi HS, Karuri D, Nyaim EO (2008). Correlation of clinical data, anatomical site and disease stage in colorectal cancer. East Afr Med J.

[13] Goodman D, Irvin TT (1993). Delay in the diagnosis and prognosis of carcinoma of the right colon. Br J Surg.

[14] Vilar E, Salazar R, Pérez-García J, Cortes J, Oberg K, Tabernero J (2007). Chemotherapy and role of the proliferation marker Ki-67 in digestive neuroendocrine tumors. Endocr Relat Cancer.

[15] Klimstra DS, Modlin IR, Adsay NV (2010). Pathology reporting of neuroendocrine tumors: application of the Delphic consensus process to the development of a minimum pathology data set. Am J Surg Pathol.

